# Evaluation of minimal residual disease using next-generation flow cytometry in patients with AL amyloidosis

**DOI:** 10.1038/s41408-018-0086-3

**Published:** 2018-05-24

**Authors:** Efstathios Kastritis, Ioannis V. Kostopoulos, Evangelos Terpos, Bruno Paiva, Despina Fotiou, Maria Gavriatopoulou, Nikolaos Kanellias, Dimitrios C. Ziogas, Maria Roussou, Magdalini Migkou, Evangelos Eleutherakis-Papaiakovou, Ioannis P. Trougakos, Ourania Tsitsilonis, Meletios A. Dimopoulos

**Affiliations:** 1 Department of Clinical Therapeutics, School of Medicine, National and Kapodistrian University of Athens, Athens, Greece; 2 Department of Biology, School of Science, National and Kapodistrian University of Athens, Athens, Greece; 3 Centro de Investigación Médica Aplicada, Clinica Universidad de Navarra, IDISNA, Pamplona, Spain

The treatment of light chain (AL) amyloidosis aims to completely eliminate the toxic light chain production, as assessed by sensitive serum- or urine-based methods such as immunofixation and free light chain (FLCs) quantification^1^. Complete hematologic responses (hemCR) can be achieved in a significant proportion of patients with AL, either with conventional therapies or with high-dose melphalan, and are associated with better overall survival and improved organ function. However, hematologic relapses still occur and organ function may continue to deteriorate due to small residual clones that may lead to disease recurrence and/or may produce very small amounts of toxic light chains which are undetectable by conventional techniques. Next-generation flow cytometry (NGF) is a very sensitive method for the evaluation of minimal residual disease (MRD) and one of the standard methods for the assessment of MRD in patients with multiple myeloma (MM), reflected in the new response assessment criteria^2^. Patients with MM who are negative for MRD have significantly improved progression-free and overall survival, even among those who have achieved a CR^3,4^. Such data are sparse in patients with AL amyloidosis, although the presence of MRD may prove a crucial factor for delayed organ response or deterioration of organ function despite conventional hemCR. The aim of the current study was to evaluate feasibility and applicability of MRD by NGF in patients with AL at hemCR.

We evaluated the presence of MRD in 20 patients with AL amyloidosis who had achieved a CR, based on negative serum and urine immunofixation, a normal FLC ratio with FLCs within normal range and a negative bone marrow (BM) biopsy^5^. We also evaluated five patients with normal FLCs but positive serum or urine immunofixation (i.e., at very good partial response, VGPR).

MRD was assessed in BM samples according to the Euroflow guidelines. In particular, bulk lysis was used for the osmotic lysis of erythrocytes and nucleated cells acquired were labeled using two independent eight-color panels, both containing CD19-PECy7, CD27-BV510, CD38-FITC, CD45-PerCPCy5.5, CD56-PE, and CD138-BV421, with additionally CD117-APC and CD81-APCC750 in the surface tube or CyIgκ-APC and CyIgλ-APCC750 in the intracytoplasmic tube. Labeled antibodies were purchased from Cytognos S.L. (Salamanca, Spain), BD Biosciences (NJ, USA), and BioLegend Inc. (CA, USA). A median number of 5 million events (range: 3.9 × 10^6^–6.1 × 10^6^) were acquired for each tube in a BD FACSCantoII cytometer and data analysis was conducted with Infinicyt software (Cytognos) that allowed merging of the two panels based on the six backbone markers. Therefore, aberrant plasma cells could be distinguished out of ~10 million evaluable cells per patient offering a highly sensitive approach for MRD detection, with median sensitivity level 2.3 × 10^−6^ (range: 2 × 10^−6^–3.1 × 10^-6^). Deploying the multiparameter nature of this assay, we confirmed the presence of all major BM subsets in all samples analyzed, thus excluding potential false-negative results due to hemodilution.

The median age of the patients at the time of diagnosis was 59 years (range: 42–75), 74% had lambda light chain AL, 90% had renal, 15% had liver, and 35% had cardiac involvement; 40% were Mayo stage-1, 50% stage-2, and 10% stage-3. At the time of diagnosis, median dFLC was 93 mg/l (range: 17–879) and four (20%) had negative serum and urine immunofixation; median BM infiltration by clonal plasma cells was 8%. Primary treatment was bortezomib-based in 85% and melphalan/dexamethasone (MDex) in 15%, while 40% have received high-dose melphalan with ASCT as consolidation. At the time of MRD testing, 9/18 (50%) patients had achieved a renal response, 4/7 (57%) patients with cardiac involvement had a cardiac response, and 2/3 (67%) a liver response.

Eight (40%) patients were negative for the presence of MRD (MRD^neg^) and 12 (60%) were positive (MRD^pos^). Notably, 5/12 (42%) MRD^pos^ cases had a very low residual tumor load at level <3 × 10^−5^ (in two cases aberrant cells were detected at levels between 10^−5^ and 10^−6^). The median time from hemCR to MRD testing was 36 months (39 months for MRD^pos^ vs 35 months for MRD^neg^ patients). MRD was positive in 3/4 patients who had negative immunofixation in the serum and urine and in 2/3 patients with dFLC <40 mg/l at the time of diagnosis. In contrast all patients in VGPR that were tested were MRD^pos^.

Organ responses of at least one involved organ were documented in 14/20 (70%) patients in CR and in particular in 8/12 (67%) of patients with MRD^pos^ vs 6/8 (75%) of patients with MRD^neg^ disease. Among cardiac responders (*n* = 4), three were MRD^neg^ and one was MRD^pos^. Renal responses were 6/9 (67%) in MRD^pos^ and 6/8 (75%) in MRD^neg^ patients. Among MRD^neg^ patients, 3/8 had response in more than one organ (both had a cardiac and a renal response); among MRD^pos^ patients all had responses to a single organ.

We then analyzed for possible differences in the baseline characteristics of those in CR that achieved vs those that did not achieve MRD negativity; we found no significant differences in baseline characteristics such as age, gender, serum FLC levels or dFLC, BM infiltration by plasma cells, NTproBNP levels, or Mayo stage (all *p* values >0.5). Among patients who received ASCT, 2/8 (25%) were MRD^neg^ vs 6/12 (50%) MRD^neg^ among patients who did not have ASCT as part of their primary therapy (*p* = 0.264). Of the three patients treated with MDex, one (33%) was found MRD^neg^. A summary of all available baseline characteristics in MRD^pos^ vs MRD^neg^ patients of our cohort are provided in Table 1. Thus, we could not identify possible baseline factors associated with a higher probability of MRD^neg^, in this highly selected population that includes patients with low or intermediate risk disease, who achieved a hemCR and who have already had a long survival associated with high rates of organ responses.

**Table 1:** Baseline characteristics of AL patients in hemCR that were tested for MRD

	All patients (*N* = 20)	MRD^neg^ (*N* = 8)	MRD^pos^ (*N* = 12)
Age	59 (42–75)	57 (46–70)	60 (42–72)
Male/female	7/13	3/5	4/8
eGFR ml/min/1.73 m^2^	93 (9->140)	77	107
eGFr <50 ml/min/1.73 m^2^	5	3	2
Renal involvement	18 (90%)	8 (100%)	10 (83%)
Proteinuria gr/24 h	7.3 (4–22)	9.3 (5.7–22)	7 (4–12)
dFLC (median/range)	93 (17–879)	202 (17–795)	68 (18–879)
Cardiac involvement	7	3	4
NTproBNP (pg/ml)	550 (30–4396)	796 (87–3415)	346 (30–4396)
Mayo stage 1/2/3	8/10/2	3/4/1	5/6/1
dFLC <40 mg/l	3	1/3 (33%)	2/3 (67%)
BM infiltration	8% (0–30%)	5% (0–20)	15% (0–30)
Bortezomib-based induction	17 (85%)	7	10
MDex	3 (15%)	1	2
Consolidation with HDM/ASCT	8 (40%)	2/8 (25%)	6/8 (75%)
Any organ response	14 (70%)	6/8 (75%)	8/12 (67%)
Renal response	12/18 (67%)	6/8 (75%)	6/10 (60%)
Cardiac response	4/7	3/3 (100%)	1/4 (25%)
Liver response	2/3	1/1 (100%)	1/2 (50%)

**Fig. 1:**
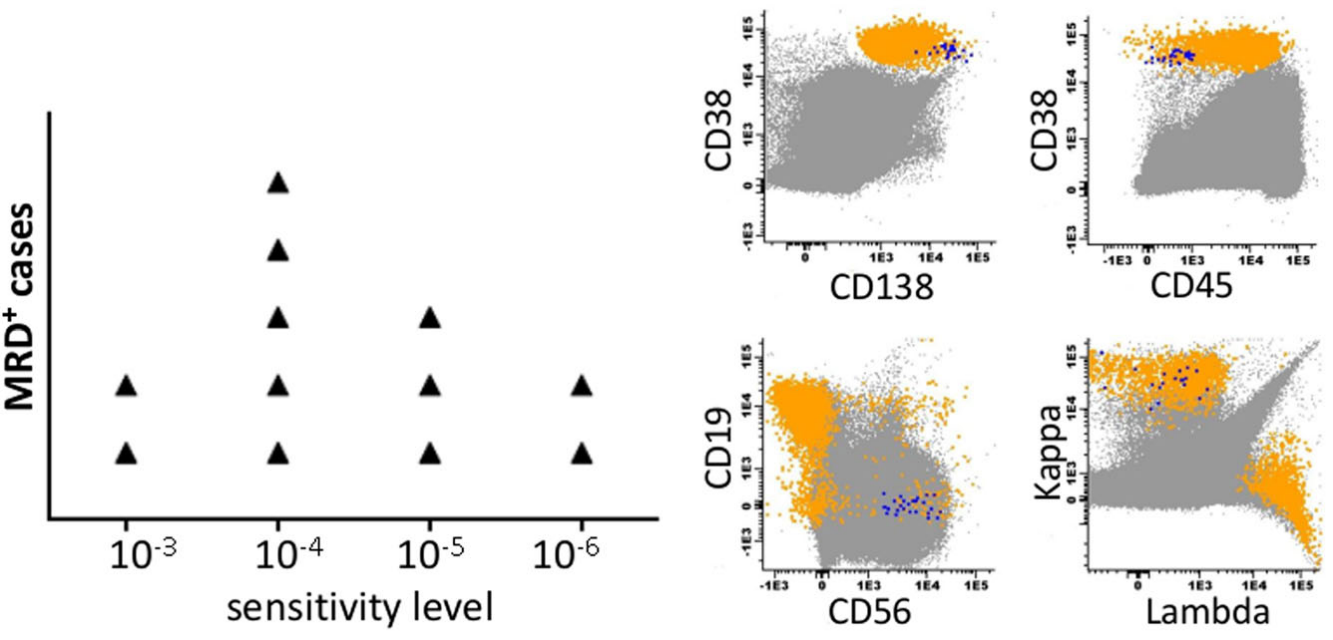
MRD detection of AL patients using next-generation flow cytometry (NGF). Left panel: The 12 MRD + AL cases of our cohort according to the detection level of aberrant plasma cells (APCs). Right panel: A phenotypic analysis of MRD + case at the level of 10^-6^. APCs (showing in blue) have a clearly distinct phenotype from normal plasma cells (shown in orange). The rest of the bone marrow nucleated cells are shown in gray

This is the first report on MRD evaluation in patients with AL amyloidosis, using NGF, with high sensitivity levels approaching 10^−6^. Importantly, in 5/12 MRD^pos^ cases residual clonal plasma cells would have been undetectable if a lower level of sensitivity had been used (Fig. 1) and as a consequence, these patients would have been considered as MRD^neg^, indicating the importance of high sensitivity methods for MRD assessment. It is also important that among patients with very low levels of detectable clonal light chains at the time of diagnosis we were able to identify MRD after treatment. Thus, NGF may also be a useful method for the detection of clonal disease in patients with very low burden of light chains which may be difficult to detect but which may also be quite toxic, as suggested by previous studies using less-sensitive flow cytometry methods^6,7^.

In AL amyloidosis the plasma cell clone is usually modest in size and usually lacks high-risk cytogenetics such as del17p or t(4;14)^8,9^. Treatment with bortezomib-based therapy or with high or conventional dose melphalan results in high response rates and deep responses. Thus, despite the high depth of detecting aberrant plasma cells in our setting, it would be expected that more patients in hemCR would be MRD^neg^. Our results, however, indicate that more than half of AL patients in hemCR (by conventional methods) are MRD positive (60%). The implications of this observation may be significant, especially if confirmed in larger series. The presence of MRD may be associated with a higher chance of hematologic relapse and subsequent organ function deterioration, necessitating therapy. Moreover, in MRD-positive patients the minimal amount of the toxic light chains produced by residual clonal cells may delay or undermine the restoration of organ function, or, may lead to further organ function deterioration. Conversely, MRD negativity may be associated with deeper organ responses, with responses in more “sensitive” organs, such as the heart, and with responses in more than one organ. The small number of patients in our study does not allow for firm conclusions however, it is notable that among cardiac responders, 3/4 were MRD negative. Another aspect underscored by our results is the relatively low incidence of MRD negativity among patients who had ASCT, which highlights concerns about the need of further consolidation or maintenance after ASCT in patients with AL, similar to myeloma. Similarly, a recent study by Lee et al.^10^ reported that 2/5 (40%) AL amyloid patients after ASCT were MRD^neg^. The introduction of novel strategies such as monoclonal antibodies targeting CD38 (such as daratumumab^11–13^) or those targeting the amyloid fibrils^14^ will change the potential options for patients that remain MRD positive.

In conclusion, among patients with AL amyloidosis in sustained hemCR, 40% were MRD^neg^ and 60% were MRD^pos^, as assessed with high sensitivity NGF. These findings may have implications in the management of patients with AL who achieve a hemCR, especially for patients who fail to achieve an organ response and may also have implications for their management, in an era of expanding treatment options.
